# Promoting Physical Activity in Japanese Older Adults Using a Social Pervasive Game: Randomized Controlled Trial

**DOI:** 10.2196/16458

**Published:** 2021-01-06

**Authors:** Luciano Henrique De Oliveira Santos, Kazuya Okamoto, Ryo Otsuki, Shusuke Hiragi, Goshiro Yamamoto, Osamu Sugiyama, Tomoki Aoyama, Tomohiro Kuroda

**Affiliations:** 1 Department of Social Informatics Graduate School of Informatics Kyoto University Kyoto Japan; 2 Graduate School of Medicine Kyoto University Kyoto Japan; 3 Division of Medical Information Technology and Administration Planning Kyoto University Hospital Kyoto Japan; 4 Department of Real World Data Research and Development Graduate School of Medicine Kyoto University Kyoto Japan

**Keywords:** aged, physical activity, pervasive games, social interaction

## Abstract

**Background:**

Pervasive games aim to create more fun and engaging experiences by mixing elements from the real world into the game world. Because they intermingle with players’ lives and naturally promote more casual gameplay, they could be a powerful strategy to stimulate physical activity among older adults. However, to use these games more effectively, it is necessary to understand how design elements of the game affect player behavior.

**Objective:**

The aim of this study was to evaluate how the presence of a specific design element, namely social interaction, would affect levels of physical activity.

**Methods:**

Participants were recruited offline and randomly assigned to control and intervention groups in a single-blind design. Over 4 weeks, two variations of the same pervasive game were compared: with social interaction (intervention group) and with no social interaction (control group). In both versions, players had to walk to physical locations and collect virtual cards, but the social interaction version allowed people to collaborate to obtain more cards. Changes in the weekly step counts were used to evaluate the effect on each group, and the number of places visited was used as an indicator of play activity.

**Results:**

A total of 20 participants were recruited (no social interaction group, n=10; social interaction group, n=10); 18 participants remained active until the end of the study (no social interaction group, n=9; social interaction group, n=9). Step counts during the first week were used as the baseline level of physical activity (no social interaction group: mean 46,697.2, SE 7905.4; social interaction group: mean 45,967.3, SE 8260.7). For the subsequent weeks, changes to individual baseline values (absolute/proportional) for the no social interaction group were as follows: 1583.3 (SE 3108.3)/4.6% (SE 7.2%) (week 2), 591.5 (SE 2414.5)/2.4% (SE 4.7%) (week 3), and −1041.8 (SE 1992.7)/0.6% (SE 4.4%) (week 4). For the social interaction group, changes to individual baseline values were as follows: 11520.0 (SE 3941.5)/28.0% (SE 8.7%) (week 2), 9567.3 (SE 2631.5)/23.0% (SE 5.1%) (week 3), and 7648.7 (SE 3900.9)/13.9% (SE 8.0%) (week 4). The result of the analysis of the group effect was significant (absolute change: η^2^=0.31, *P*=.04; proportional change: η^2^=0.30, *P*=.03). Correlations between both absolute and proportional change and the play activity were significant (absolute change: *r*=0.59, 95% CI 0.32 to 0.77; proportional change: *r*=0.39, 95% CI 0.08 to 0.64).

**Conclusions:**

The presence of social interaction design elements in pervasive games appears to have a positive effect on levels of physical activity.

**Trial Registration:**

Japan Medical Association Clinical Trial Registration Number JMA-IIA00314; https://tinyurl.com/y5nh6ylr (Archived by WebCite at http://www.webcitation.org/761a6MVAy)

## Introduction

The proportion of elderly people is increasing in populations worldwide as a natural consequence of the progression in health care and technology, as well as decreased birth rates [1]. This phenomenon intensifies the need for effective strategies to promote the well-being of elderly people. Previous work addressed that goal using different approaches, each with different levels of success [2]. Electronic games became a major topic of interest, being used in varied fields such as the rehabilitation of psychomotor functions [3,4], prevention of age-related diseases [5], and promotion of active lifestyles [6]. In that context, pervasive games are a relatively recent field of research [7].

A pervasive game is an electronic game that blends the real and virtual worlds, proposing interactions that incorporate elements from players’ daily lives into the game rules [8]. They can be especially beneficial for elderly people for three main reasons. First, such games often invite players to walk to real-world locations [9] or to perform different exercises with their bodies [10], and both types of interaction promote higher levels of physical activity, a practice strongly correlated with well-being among the elderly [11-13]. Secondly, these games can encourage social connection, which is an important factor in the maintenance of both physical and mental health among senior citizens [14]. Finally, pervasive gaming experiences also focus on casual gameplay by nature, allowing players to learn on their own pace and giving them a lot of freedom to choose how much they want to engage with the game, an aspect of particular interest for elderly players [15].

In this work, we explore pervasive games for elderly individuals from a design perspective, focusing on a specific design aspect—social interaction—and targeting a specific goal, which is to promote physical activity. To do that, we used a previously developed pervasive mobile game [16-18] and performed a randomized controlled trial with Japanese elderly people, evaluating whether the presence of social interaction elements has any effect on levels of physical activity.

## Methods

### Design

A single-blind randomized controlled trial was conducted to compare two groups. In the intervention group, participants played a version of a pervasive game that included social interaction elements. In the control group, participants played a single-player version of the same game. A 4-week protocol was adopted. During the first week, participants did not play the game, but their level of physical activity was measured to serve as the baseline. During the remaining weeks, participants could freely play the game, while their levels of physical activity and game actions were monitored. Participants were blinded to group assignment, but researchers were not.

### Participants

Participants were recruited from community-dwelling senior adults living in Kyoto City, Japan, using flyers. Because this research is contextualized as a preventive health intervention and it is expected that experience with games will become increasingly common among older adults in the future, we adopted a broader age range of 50 years and older into the study’s inclusion criteria, aiming for middle-aged and older adults. Additional criteria included healthy people with independent ambulation and no cognitive or physical impairment that prevented them from understanding the instructions of the game or taking short walks.

All participants signed an informed consent term, and the research protocol was approved by the Kyoto University Hospital’s Ethical Committee, which reports compliance with the Declaration of Helsinki. Participants were offered a shopping voucher worth 10,000 Japanese yen (US $96.51) as compensation for their participation in the study. The only requirement to receive the voucher was answering the final questionnaire at the end of the study.

### Game

Participants played a pervasive location-based mobile game called Shinpo, which, in free translation, means “steps of the gods” in Japanese. This game was first evaluated for its feasibility in Kyoto [19] and later used in a similar study in Brasília, Brazil [20].

**Figure 1:**
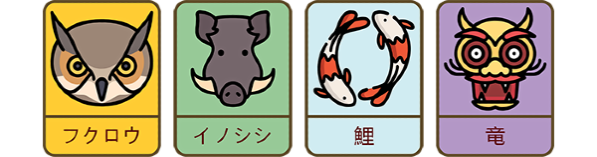
Examples of virtual cards in Shinpo.

In Shinpo, players must collect virtual cards (Figure 1) by visiting shrines and temples in Kyoto city. Each card features an animal and a color, which indicates its level. There are four levels, and the goal of the game is to collect the card of highest level for all animals.

**Figure 2:**
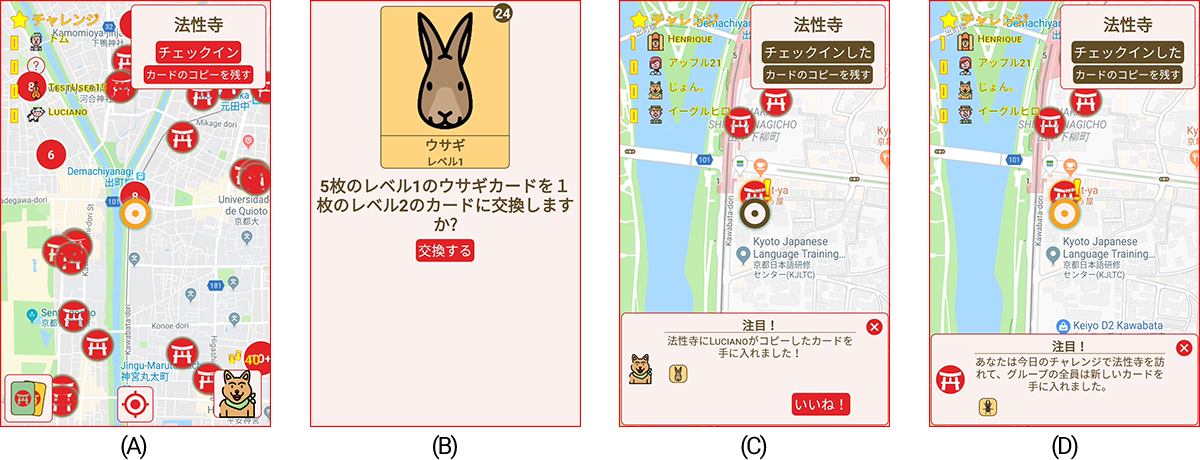
Examples of game screens. (A) Players used the map to search their neighborhood to find temples and shrines. (B) Players could visualize their cards and exchange 5 equal cards of a given level for 1 card of the next level. (C) Players could leave a copy of a card in a place they visited, and other players would see a mark in places where this was done. (D) Players were assigned to challenge groups and were notified when members of the group visited places, which added cards to all members of the group.

On the main screen of the game (Figure 2A), players could see a map showing their current location and nearby hotspots, which were indicated by red icons. Hotspots were defined using Google Maps information about shrines and temples in Kyoto City. After visualizing nearby hotspots, players had to walk physically toward them, and when they were within 50 m of their locations, a “check-in” option appeared, allowing the player to register a visit. For safety reasons, players were not expected to keep the game screen open while walking and should have accessed the game only when they arrived at a destination. If the game was open and the player’s speed exceeded a certain threshold, the game warned the player not to walk while looking at the smartphone.

A player received a random level 1 card for every unique hotspot they visited within a day, or for every 1000 steps they walked. The step count was measured using a background service that operated whenever the smartphone was turned on. This software used Google Android’s Sensor application programming interface, which is the same as that of Google Fit, an application previously shown to have an accuracy comparable to that of wearable devices [21]. At any moment, players could trade 5 equal cards of the same level for 1 card of the next level (Figure 2B).

In the version of the game with social interaction, additional rules were added to stimulate players to interact and collaborate to obtain more cards:

- Players could leave one copy of any of their cards, up to level 2, on each hotspot they visited each day, once in the morning and once in the afternoon (Figure 2C). If this happened, that hotspot would be highlighted on other players’ devices, with an exclamation mark added to its icon. When a different player visited the highlighted hotspot, they received a copy of the card left there and the original visitor received additional random cards.
- Every day, players were randomly assigned to a challenge group, and anytime a person in the group collected a card, all other members also received a card. The members of the group and their contribution to the challenge were shown to all other members to promote a sense of unity (Figure 2D).
- All players could choose a public avatar and nickname and could make a short self-introduction; when players received cards as the result of other players’ actions, they had a chance to give them a “like” (Figure 2D).

The feasibility study and follow-up evaluations [17,22] suggested that these mechanics allowed players to feel more engaged in playing the game by working together with other people. We hypothesized that this setup would result in a greater positive effect on levels of physical activity.

### Outcome Measures

The main observed outcome was the level of physical activity, measured by the average number of steps per week, in a 4-week period. During the first week, participants did not play the game, but their step count was still monitored. This monitoring was performed to assess their baseline level of physical activity. After that, they played the game for 3 weeks. Weekly cycles were chosen to consider different lifestyle behavior patterns during different days of the week. Also, to account for individual differences among participants, the proportional change in relation to the individual’s baseline level of physical activity was also analyzed.

To evaluate how much participants played the game, the weekly average number of visits to hotspots was also observed. Within a single day, this observation represented the number of unique hotspots visited by the player, while within a week, it was the sum of the visits in each day of the week (ie, the same hotspot was not counted twice for the same day, but it could be counted twice in a week). This measurement was used because players were directed to not keep the game open while walking, so play time was not a good measurement of how much a person played.

As a secondary evaluation, participants were also asked to answer a final survey composed of the Game Experience Questionnaire (GEQ) [23] (including the “Social presence” section) and the System Usability Scale (SUS) [24], since these questionnaires have been widely used in previous works. The items of these questionnaires were translated and cross-verified by 2 Japanese native speakers. All items consisted of 5-point Likert scales. In the case of the GEQ, the scale was 0=didn’t feel it at all to 4=felt it extremely; for the SUS, the scale was 0=don’t agree at all to 4=completely agree. Besides these two metrics, participants were also asked to respond to the statement “I remembered to carry the smartphone with me when I left home” according to the following scale: 0=every day, 1=often, 2=sometimes, 3=almost never, and 4=never. Finally, at the end of the survey, participants could freely write comments, criticisms, or suggestions.

### Procedures

At the beginning of the study, participants signed an informed consent form and completed the first questionnaire (ie, regarding their previous experience with games and technology). Their smartphones were checked for compatibility, and the game was then installed. Compatible systems included Android-based smartphones with Android operating system version 6.0 or above with a global positioning systems (GPS) sensor and an internet connection. Participants who did not have a compatible smartphone or who could not or did not want to use their personal devices were lent a previously prepared one by the researchers. Participants were given an instruction manual and an oral presentation about basic smartphone usage and the rules and interface of the game.

Participants were randomly assigned to either the social interaction group (intervention group) or no social interaction group (control group), and remained blind to their group assignment. During the first week, no participant could play the game, and the application only showed a message indicating that their step count was being monitored and providing the date on which the game would become available. From the second week on, the game became available and participants were able to play freely. There was a follow-up meeting on the same weekday every week, in which researchers were available to clarify any doubts or solve technical problems.

Participants were instructed about the importance of keeping their smartphones turned on and carrying them with them whenever they left their homes, throughout the duration of the study, since they would be used to measure the participants’ step counts. In the final survey, they were also asked about their compliance with these instructions.

After the end of the trial, participants answered the final questionnaire to evaluate their experience while playing and received the voucher for 10,000 Japanese yen (US $96.51).

All questionnaires were administered by researchers, who were available to answer any questions about the items.

### Data Analysis

Questionnaire data were consolidated to report percentages for each item, while means and standard errors were calculated for demographic data. Participants were considered active in a given day if they performed at least one check-in at a hotspot during that day. If a participant spent 3 or more days being inactive in a week, they were considered an inactive participant for that week. No participant explicitly asked to leave the study. Data were processed using Python (mainly the Pandas and Matplotlib packages) and exported into a suitable format for R, which was used for statistical analysis.

To analyze the effects, we calculated the change in the number of steps for each week from the baseline values in week 1. These measurements were made for each participant in relation to their own individual baseline value, and the proportional change was also calculated (ie, the absolute change divided by the baseline value). This was done to consider the effect on each participant in relation to their own initial baseline value.

In the statistical model, the change for each week after baseline was considered to be a repeated measure, and an analysis of variance (ANOVA) was performed—with group and week as factors—for participants who remained active until the end of the experiment. The relationship between the change in the step count and the number of hotspot visits was evaluated using Pearson’s correlation coefficient (*r*).

For the final questionnaires, the value of negatively phrased items was inverted, and the mean and standard error was calculated for each category of the GEQ and for the SUS as a whole, resulting in scores that ranged from 0 to 4.

## Results

### Participants

A total of 20 participants (16 females, 4 males) were recruited, and 18 participants (14 females, 4 males) remained active until the end of the study. Table 1 presents basic information about the participants, including their previous experience with games and technology.

**Table 1:** Basic data of study participants (N=20).

		Baseline		Active until the end	
Participant data		No social interaction	Social interaction	No social interaction	Social interaction
**Demographics**					
	Participants, n	10	10	9	9
	Sex (female), n (%)	9 (90)	9 (90)	7 (78)	7 (78)
	Age (years), mean (SE)	66.4 (10.2)	63.2 (8.5)	64.6 (8.9)	62.2 (8.4)
**PC^a^ usage frequency, n (%)**					
	Never	3 (30)	2 (20)	3 (33)	2 (22)
	Approximately 1 time/week	1 (10)	3 (30)	1 (11)	3 (33)
	≥2 times/week	2 (20)	2 (20)	1 (11)	2 (22)
	Daily	4 (40)	3 (30)	4 (44)	2 (22)
**PC activity^b^, n (%)**					
	Use email	7 (70)	5 (50)	6 (67)	4 (44)
	Browse the web	4 (40)	6 (60)	4 (44)	5 (56)
	Search the web	4 (40)	6 (60)	4 (44)	5 (56)
	Read the news	5 (50)	5 (50)	4 (44)	4 (44)
	Use social networks^c^	3 (30)	3 (30)	4 (44)	2 (22)
	Manipulate photos	5 (50)	5 (50)	4 (44)	4 (44)
	Use office applications	6 (60)	6 (60)	5 (56)	5 (56)
**Mobile phone activity^b^, n (%)**					
	Make calls	9 (90)	10 (100)	8 (89)	9 (100)
	Use email	9 (90)	10 (100)	8 (89)	9 (100)
	Browse the web	7 (70)	9 (90)	6 (67)	8 (89)
	Use social networks	7 (70)	10 (100)	6 (67)	9 (100)
	Install and use apps	7 (70)	10 (100)	6 (67)	9 (100)
**Play frequency of nonelectronic games, n (%)**					
	Never	0 (0)	5 (50)	0 (0)	5 (50)
	Very rarely	7 (70)	4 (40)	6 (60)	3 (30)
	Approximately 1 time/week	2 (20)	0 (0)	2 (20)	0 (0)
	≥2 times/week	1 (10)	1 (10)	1 (10)	1 (10)
	Daily	0 (0)	0 (0)	0 (0)	0 (0)
**Play frequency of electronic games, n (%)**					
	Never	1 (10)	3 (30)	1 (10)	3 (30)
	Very rarely	6 (60)	2 (20)	5 (50)	2 (20)
	Approximately 1 time/week	0 (0)	0 (0)	0 (0)	0 (0)
	≥2 times/week	0 (0)	3 (30)	0 (0)	2 (20)
	Daily	3 (30)	2 (20)	3 (30)	2 (10)
**Devices used to play^b,d,e^, n (%)**					
	PC	3 (33)	2 (29)	3 (38)	1 (17)
	Portable console^f^	1 (11)	1 (14)	1 (13)	1 (17)
	Conventional console^g^	0 (0)	0 (0)	0 (0)	0 (0)
	Mobile phone or tablet	5 (56)	5 (71)	5 (63)	5 (83)
**Play partners^b,d,e^, n (%)**					
	Young family members	1 (11)	0 (0)	1 (13)	0 (0)
	Adult family members	0 (0)	0 (0)	0 (0)	0 (0)
	Friends	1 (11)	0 (0)	1 (13)	0 (0)
	Strangers online	1 (11)	0 (0)	1 (13)	0 (0)

^a^Personal computer.

^b^Participants could indicate more than one item.

^c^LINE, Facebook, Twitter, Instagram, etc.

^d^Some people reported playing but did not indicate an option for this item.

^e^Percentages are relative to the number of people who reported any play activity.

^f^Nintendo 3DS, PlayStation Portable, etc.

^g^Nintendo Switch, PlayStation, etc.

Participants in both groups had similar backgrounds of experience using a personal computer (PC), but there were some differences in other areas. For mobile phone usage, participants in the social interaction group had a higher level of self-reported skill, although both groups reported a high level. As for previous experience with games, more participants in the no social interaction group reported playing nonelectronic games than participants in the social interaction group, while the opposite was true for electronic games. Only participants in the no social interaction group reported ever playing games together with other people.

Participants could freely write down titles of games that they often played. For nonelectronic games, cited titles included the following (stated as number of citations by participants in the no social interaction group and in the social interaction group, respectively): crosswords (2 and 0), card games (1 and 1), sudoku (1 and 1), Go (1 and 1), puzzle games (1 and 0), Shogi (0 and 1), Reversi (0 and 1), and Concentration (0 and 1). For electronic games, cited titles included the following: Disney Tsum Tsum (1 and 4), Pokémon (1 and 0), Pokémon Let’s GO (1 and 1), Pokémon GO (1 and 2), Animal Crossing (several versions) (2 and 1), Solitaire (1 and 1), Splatoon (0 and 1), Homescapes (1 and 0), the Legend of Zelda (1 and 0), Monster Hunter (1 and 0), puzzle games (1 and 0), Everybody’s Golf (0 and 1), Mahjong (0 and 1), and LINE POP (0 and 1).

Except for 1 participant in the social interaction group, all participants used borrowed devices. Of the 20 total participants, 9 had an iPhone (Apple Inc) and could not install the application, while the remaining 10 participants that borrowed a mobile phone said that they did not want to install the application on their own device.

### Main Outcome

Step count data are shown in Table 2 for participants who remained active until the end of the experiment. The baseline column shows the step counts recorded during the first week for each group. The remaining columns report the change for the subsequent weeks in relation to the participants’ own baseline values. If w_p,i_ is the number of steps on week “i” for participant p, and b_p_ is the baseline number of steps for that participant, then the absolute change is calculated as ∆_p,i_=w_p,i_–b_p_ and the proportional change is calculated as q_p,i_=∆_p,i_/b_p_.

For the absolute change, the effect of group as a factor in the ANOVA was significant (*P*=.04; η^2^=0.31). No relevant relationship was found with week as a factor (*P*=.19). For proportional change, using group as a factor resulted in a *P* value of .03 (η^2^=0.30), while using week as a factor resulted in a *P* value of .17.

For number of hotspot visits, the no social interaction group had a mean of 42.8 (SE 15.8) visits in week 2, 89.7 (SE 24.5) visits in week 3, and 96.2 (SE 35.4) visits in week 4. By comparison, the social interaction group had a mean of 169.0 (SE 63.9) visits in week 2, 115.0 (SE 40.0) visits in week 3, and 140.0 (SE 57.6) visits in week 4.

The correlation analysis between the absolute change in the number of steps and the number of hotspot visits resulted in a correlation factor of *r*=0.59 (95% CI 0.32 to 0.77). When proportional change was considered, the correlation factor was *r*=0.39 (95% CI 0.08 to 0.64).

**Table 2:** Number of steps at baseline for each group and individual variations in subsequent weeks.

			Median change (min; max)		
Group and change from baseline		Baseline, median (min; max)	Week 2	Week 3	Week 4
**No social interaction group**					
	Absolute change^a^	42,305 (27,687; 71,028)	921 (–7986; 11,454)	437 (–7999; 8587)	–1143 (–6611; 6476)
	Proportional change (%)^b^	NA^c^	4.8 (–16.0; 28.1)	1.8 (–11.3; 16.3)	–1.6 (–9.7; 18.7)
**Social interaction group**					
	Absolute change	51,254 (20,356; 65,594)	10,494 (–508; 27,167)	5954 (4944; 20,711)	3722 (–2691; 22,891)
	Proportional change (%)	NA	24.4 (–1.0; 54.5)	24.2 (7.6; 41.5)	10.0 (–12.2; 45.9)

^a^Absolute values indicate the change in the weekly number of steps when compared with the user’s own baseline value.

^b^Proportional values indicate the absolute value divided by the user’s own baseline value (ie, if ∆_p,i_ is the change from baseline to week “i” for participant p, and b_p_ is the baseline number of steps for that participant, then the proportional change q_p,i_=∆_p,i_/b_p_.

^c^NA: not applicable.

### Game Experience

The scores for the usability and game experience questionnaires are summarized in Table 3. The scores of component items ranged from 0 to 4; therefore, a value of 2 or greater indicates a high valuation. The data included are only those from participants who stayed active until the end of the experiment. Since these data were used only as complementary information for future interventions, no further statistical analysis was performed.

For the statement “I remembered to carry the smartphone with me when I left home,” 8 participants in the no social interaction group responded “everyday,” and 1 participant in that group responded “often.” In the social interaction group, all participants responded “everyday.”

Participants’ comments about the game were summarized by one of the researchers who is a native Japanese speaker and briefly interacted with the participants at the last meeting, in which they completed the final questionnaire. Comments made during the last meeting and also written on the questionnaires included the following: “The accuracy of the GPS could be improved,” “I enjoyed visiting new places,” “I want to continue playing,” “I want Shinpo to notify me when I am near the temple,” “It is difficult to understand the communication function,” “I think it would have been more interesting if I was used to using smartphones,” and, “There were too many notifications.”

**Table 3:** Mean scores for Game Experience Questionnaire (GEQ) and System Usability Scale (N=18).

		Mean score (SE)^a^	
Category		No social interaction group (n=9)	Social interaction group (n=9)
**GEQ: Core**			
	Competence	1.6 (0.3)	2.1 (0.3)
	Sensory and imaginative immersion	1.7 (0.4)	2.3 (0.3)
	Flow	1.4 (0.4)	1.6 (0.3)
	Tension	0.5 (0.2)	1.1 (0.3)
	Challenge	1.3 (0.3)	1.5 (0.3)
	Negative affect	0.7 (0.3)	1.0 (0.2)
	Positive affect	2.6 (0.4)	3.0 (0.3)
**GEQ: Social presence**			
	Psychological involvement: empathy	0.8 (0.2)	2.0 (0.4)
	Psychological involvement: negative feelings	0.5 (0.2)	1.0 (0.3)
	Behavioral involvement	0.5 (0.3)	1.5 (0.5)
System Usability Scale		2.6 (0.2)	2.8 (0.1)

^a^Scores are normalized in the 0 to 4 interval; values equal or greater than 2 indicate a high score for the item.

## Discussion

### Principal Results

Due to the limited number of participants, there were a few differences between the groups after randomization; however, both groups had similar previous experience with games and technology, and the proportions remained similar when only participants who remained active until the end were considered. For PC usage, most participants had at least some experience, and for smartphones, all participants knew at least the basic operations. Unsurprisingly, when previous experience with games was considered, most participants in both groups reported never playing games or playing them very rarely. Participants in both groups reported using PCs, handheld consoles, and smartphones to play, and no participant reported using a traditional gaming console. Most people reported playing alone, with only a few people in the no social interaction group and no one from the social interaction group reporting playing with other people.

For the main outcome, a greater positive effect was observed in the social interaction group than in the no social interaction group. The statistical analysis with respect to the absolute change indicated a large effect size (η^2^=0.31), and *P*=.04 indicates a statistically significant difference. For proportional change, similar results were observed, with *P*=.03 (η^2^=0.30). The correlation between the number of hotspot visits and the effect was also statistically significant, with a positive value in both cases (absolute change: *r*=0.59, 95% CI 0.32 to 0.77; proportional change: *r*=0.39, 95% CI 0.08 to 0.64), which suggests that the gameplay was one of the main factors that generated the effect. These results lead us to believe that the social interaction elements had a relevant positive effect on the levels of physical activity of the players.

The evaluations of game experience and system usability were used as complementary information only, and statistical analysis was not performed. The mean scores were higher for all items in the social interaction group. This suggests that this version of the game had a greater effect on players in “positive” aspects, such as “competence” and “sensory and imaginative immersion,” but also in “negative” ones, such as “tension” and “psychological involvement—negative feelings.” A possible explanation is that this version of the game provided a more intense experience in general. Nonetheless, if we consider as “positive” the categories of “competence,” “sensory and imaginative immersion,” “flow,” “positive affect,” “psychological involvement—empathy,” and “behavioral involvement,” the social interaction version was highly evaluated in 4 of them, and in none of the “neutral” or “negative” categories. For usability, both versions were highly evaluated.

### Limitations

The limitations of this study are as follows. The main outcome was measured using smartphone software. The methodology has been evaluated in previous studies, and the authors of those studies concluded that it is adequate; however, the comparative studies considered indoor environments, and outdoor measurements may render different results. In addition, there might be differences in accuracy for different age groups. In this study, we considered weekly cycles and also analyzed the proportional values to evaluate the effects for each individual participant.

Future interventions might test similar settings with a different device, such as an external pedometer, and compare the results. In both cases, because the data are collected not in a controlled environment but rather in a user-dependent context and participants’ adherence to carrying the device is self-reported, there might be inaccuracies in the measurements. We specifically instructed participants about the importance of carrying the smartphones with them so that their step counts could be measured; with the exception of 1 participant who responded “often” to the statement “I remembered to carry the smartphone with me when I left home,” all other participants reported following this guideline every day. Nonetheless, future interventions might use more objective ways of measuring compliance.

Step counts were observed in a continuous state, considering any daily activity of participants, and the number of visits to hotspots was used as a proxy measurement of the amount of game play, since participants were encouraged to only open the game to check in at hotspots and close it between visits. Because step counts for the baseline week were also measured continuously and the analysis considered the observed change, the results are still relevant. Nonetheless, further interventions might also separate in-game counts explicitly and analyze if there is any difference.

The sample population was mostly composed of women, due in part to the fact that the majority of the Japanese elderly population is female, but mainly because of recruitment difficulties caused by the cultural context, such as the fact that most elderly people in Japan engage primarily in activities in gender-specific groups. A more gender-balanced sample would better represent the actual adult elderly population.

Although this study is included in the more general field of interventions to improve the quality of life of older adults, it focused specifically on increasing physical activity based on previous results that show a strong correlation between these variables. Future interventions could measure these two variables explicitly and evaluate their relationship in the context of pervasive games. Also, the questionnaires used to assess usability and game experience were not statistically validated, and were used only to provide complementary information rather than to draw conclusions about the effect. The use of validated metrics would allow us to further understand the data and test more hypotheses.

The proposed social interaction mechanics focused mainly on collaboration and virtual interaction. More types of social interaction and different variables can be tested, such as competition, direct (in-person) interaction, group dynamics, and interaction with family and friends, among others.

The final purpose of promoting physical activity is to increase the well-being of the elderly population. In this study, we evaluated specifically whether social elements in pervasive games would have any effect on the level of physical activity, but to evaluate the effect on well-being in general would require many additional metrics and a much longer period of time.

### Comparison With Prior Work

This work is a follow-up of a similar study in Brazil [20], which was performed with a version of the game that was adapted to Brazilian culture but was otherwise identical. The results found in this evaluation were stronger than the results in the Brazil study, with similar values for proportional effects (the absolute effect was also larger, but the baselines were very different) and larger effect sizes for both absolute (η^2^=0.30 versus η^2^=0.19) and proportional (η^2^=0.31 versus η^2^=0.27) measurements. Additionally, while the Brazil study was inconclusive for the correlation between hotspot visits and proportional effect, the results in this study were statistically significant in all cases.

A few studies have already employed pervasive games or gamified apps targeting older adults, usually focusing on specific goals, such as cognitive training [25] and the promotion of physical activity using social incentives [26,27]. A successful commercial example that does not target elderly adults in particular but that became extremely popular among people of all ages is Pokémon GO [28]. Different studies have analyzed its effects on levels of physical activity, finding overall positive results, especially in the first weeks of use [29-31].

Recently, new research has emerged [32,33] analyzing in greater depth the experience of elderly people in play, based on the principle that games—even serious games—should primarily be fun because health benefits come later as a natural consequence of play [34]. In that respect, a few studies have attempted to clarify elderly players’ needs and motivations and investigated possible challenges in designing games for older audiences, listing common physical and cognitive limitations that should be taken into consideration [35-39]. Other studies have attempted to identify the preferences of elderly people regarding the content and/or genre of the games [15,40-42]. In this study, we evaluated social interaction as a design element in the context of pervasive games, a new kind of game that is only now being explored. This study was limited and focused on a specific metric, namely physical activity, which was taken as a proxy, but the results suggest that this topic should be further investigated, with the consideration of additional variables related to the game experience.

### Conclusions

In this work, we investigated whether the new genre of pervasive games could be used to increase physical activity among older adults by focusing on a specific design element, namely social interaction. Our results indicated that a pervasive game using social interaction had a greater positive effect on levels of physical activity than the same game without social interaction. This study had limitations, but the results are promising, and corroborated a previous study using the same game. In future interventions, other types of social interaction and/or design elements could be evaluated, and additional variables might be considered, such as indicators of physical and psychological health, among others.

## Appendix

Multimedia Appendix 1 CONSORT-eHEALTH checklist (V 1.6.1).

## Abbreviations

ANOVA: analysis of variance
GEQ: Game Experience Questionnaire
GPS: global positioning systems
PC: personal computer
SUS: System Usability Scale
